# Psychological approaches to obesity in young adults: state of the art

**DOI:** 10.3389/fnut.2024.1328386

**Published:** 2024-02-07

**Authors:** Rafaela Alves, Hugues Petitjean, Daria Druzhinenko-Silhan

**Affiliations:** P. S. Institut, Strasbourg, France

**Keywords:** body weight, behavioral interventions, psychodynamic management, psychological strategies, obesity prevention, cognitive therapy, weight management programs, young adult wellness

## Abstract

**Background:**

Obesity has become a significant health concern among young adults aged 18–35 years. Addressing this issue is crucial, and exploring psychological treatments and perspectives specifically for this population is essential.

**Methods:**

This literature review examines psychological treatments for obesity in young adults over the past decade. It focuses on interventions and discussions particularly relevant to this age group.

**Discussion:**

Research on obesity often overlooks young adults, with most interventions primarily focusing on weight loss and neglecting emotional aspects. Cognitive-behavioral approaches are commonly used for self-regulation and motivation, but psychodynamic perspectives remain underutilized. While group-based methods lack a detailed analysis of benefits, hybrid approaches demonstrate higher engagement compared to technology-only interventions. There is a notable gap in tailoring obesity interventions to meet the unique needs of young adults during this transitional life phase. It’s imperative to shift the focus from merely weight loss to a broader consideration of psychological, emotional, and unconscious factors. Integrating group modalities with psychodynamic approaches might offer additional benefits.

**Conclusion:**

This review highlights the need for further research into the psychological well-being of young adults with obesity. A more comprehensive approach is required to address their distinct needs and psychological factors.

## 1 Introduction

In recent years, obesity has emerged as a complex and multifactorial disease with a global impact. Over the past four decades, the prevalence of obesity has significantly increased, currently reaching almost a third of the world’s population and affecting both genders and various age groups (1, 2). Due to its serious health consequences, obesity is now considered a global epidemic, commonly referred to as “globesity” (3).

Obesity is associated with a wide spectrum of comorbidities, such as type 2 diabetes, specific forms of cancer, hypertension, heart failure, stroke, and hypercholesterolemia (4–7). As a result, healthcare costs associated with obesity in the general population have significantly increased (8). Furthermore, obesity is correlated with various mental health complications, including depression, body image disorders, stress, and reduced self-esteem, all of which contribute to a diminished quality of life for individuals coping with excessive body weight (9–11). Additionally, individuals with overweight or obesity frequently encounter discrimination and social stigma, exacerbating the already daunting challenges posed by this condition (12, 13).

While dietary interventions have shown short-term effects, long-term weight loss remains a challenge. Bariatric surgery has proven effective in achieving long-term weight loss in patients with Class III obesity (formerly known as morbid obesity, BMI of ≥40 kg/m^2^), however, the procedure does not guarantee sustained weight loss. Some individuals who undergo bariatric surgery may still face weight stabilization or regain if unhealthy lifestyle habits persist (14). Obesity is a complex disease influenced by various factors, including genetic, endocrine, psychological, social, and environmental factors (7, 15). Understanding and addressing these underlying factors are crucial in developing effective interventions and strategies to tackle obesity globally.

The prevalence of overweight and obesity among emerging adults aged 18 to 25 exceeds 40% (15, 16). Moreover, recent estimates indicate that over 40% of young adults^1^ (aged 25 to 35) in the United States present overweight or obesity (17). Obesity has become a major health problem for adults between 18 and 35 years, with evidence of an increased incidence of obesity-related diseases such as Type 2 diabetes mellitus in this age group (18). Studies (19–21) have demonstrated that numerous unhealthy changes in physical activity patterns and dietary practices, which are associated with the onset of obesity, often occur during emerging adulthood.

The economic costs (22) associated with obesity in young adults are a matter of growing concern, as the increasing prevalence of obesity has had a negative impact on healthcare systems and workplace productivity. Young adults with obesity may face chronic or acute health problems, leading to frequent absences from work or school. In addition to absence, obesity can also result in reduced workplace productivity due to chronic health issues, fatigue, and low energy levels. This can lead to decreased work efficiency and, consequently, negative economic implications for employers and the overall economy.

These findings underscore the importance of addressing obesity in young adults as a public health issue and a significant economic challenge. Prevention and intervention strategies targeted at this age group can help reduce the economic costs associated with obesity and improve the health and quality of life of affected young adults.

In this sense, the importance of early identification and intervention in obesity among young adults is a topic of great importance (9, 18). Identifying the tipping point in homeostasis during young adulthood is crucial as it represents an opportune moment for change and interventions (23). Indeed, this life phase exhibits unique characteristics that render it particularly receptive to psychological transformations. It is a transition period, where individuals confront developmental challenges, identity questions, and psychological reorganizations. This period is also physiologically marked by cerebral plasticity, as the ongoing maturation of the frontal lobe and its associated executive functions during young adulthood can influence decision-making, impulse control, and emotional regulation. These cognitive developments can play a crucial role in shaping health-related behaviors, including dietary choices, levels of physical activity, and overall lifestyle habits (19).

Understanding the specific challenges that young adults face during this transitional phase allows for the development of tailored intervention strategies to mitigate the long-term health consequences associated with obesity (7, 15). During this life stage, they are exposed to various risk factors related to obesity, such as sedentary behaviors, reduced access to nutritious foods, busy routines, deterioration in sleep quality, stress resulting from the new autonomy at university, anxiety related to sexual encounters, and social pressure regarding body conformity to societal norms (19). As a result, they often report feeling anxious and depressed (19, 24, 25).

This integrative review aims to methodically survey the scope of psychological treatments extended to young adults contending with obesity, delineating the specific treatment modalities and the distinct challenges inherent to this demographic. The endeavor seeks to discern substantive contributions that can inform the enhancement of therapeutic interventions targeting this group. The review will pinpoint specific methodologies, outcomes, and theoretical underpinnings in existing studies, providing a nuanced understanding of their effectiveness and the portrayal of the young adult experience.

## 2 Review methods

The present state of the art constitutes an integrative review of the literature from the past decade within the field of psychology, focusing on the treatment of young adults suffering from obesity. This review was conducted in alignment with the PRISMA-ScR (Preferred Reporting Items for Systematic reviews and Meta-Analyses extension for Scoping Reviews) guidelines.

The choice of this population is justified not only by the factors mentioned earlier (increasing rates of obesity, economic costs, and a favorable stage for intervention) but also by the limited focus on this demographic in the existing psychological literature (31). Recognizing this gap, the current authors are involved in a research project^2^ aimed at providing discussion groups for young adults with obesity (students at the University of Strasbourg). Both the ongoing project and this present article aim to contribute to the scientific and practical understanding of this life stage.

### 2.1 Inclusion and exclusion criteria for studies in the review

This review included studies published in the last 10 years, available in English and French, with data on professional counseling by psychologists for young adults with obesity. Studies with interventions proposed or conducted by professionals from other health fields, such as nutrition and medical care, were not included.

Studies were also excluded if they did not specifically target the young adult population, i.e., children, adolescents, and adults in general, or focused solely on eating disorders without addressing obesity as a primary subject of study.

### 2.2 Search strategy and study selection

Searches were conducted using eight electronic databases: ScienceDirect, Cairn.info, Pep web, Psychoanalytic Association, PsycInfo, Scopus, PubMed and PsycARTICLES.

The process of searching for articles occurred in two stages. First a search was conducted using keywords in French and English such as “young adult,” “obesity,” “psychotherapy.”

The identified studies were screened based on title and abstract to confirm their eligibility. These articles were then classified into different categories according to their central topic of discussion: psychological factors/disorders (those that addressed obesity associated with eating and psychiatric disorders, family factors such as parent–child relationships), treatment (the focus of the discussion was on management, highlighting intervention techniques), analysis of living and context (research that contextualized obesity demographically, for example by class and gender) and research methodology (the main objective was to present a research method).

Questions emerged regarding the initially reduced number of articles specifically dealing with psychological treatment (only 7 in total). Taking this into account, a second search was conducted using keywords in French and English such as “young adult,” “obesity,” “psychological treatment,” “support group,” “psychotherapeutic support,” to explore the possibility of finding additional articles on management.

## 3 Results

**Figure 1:**
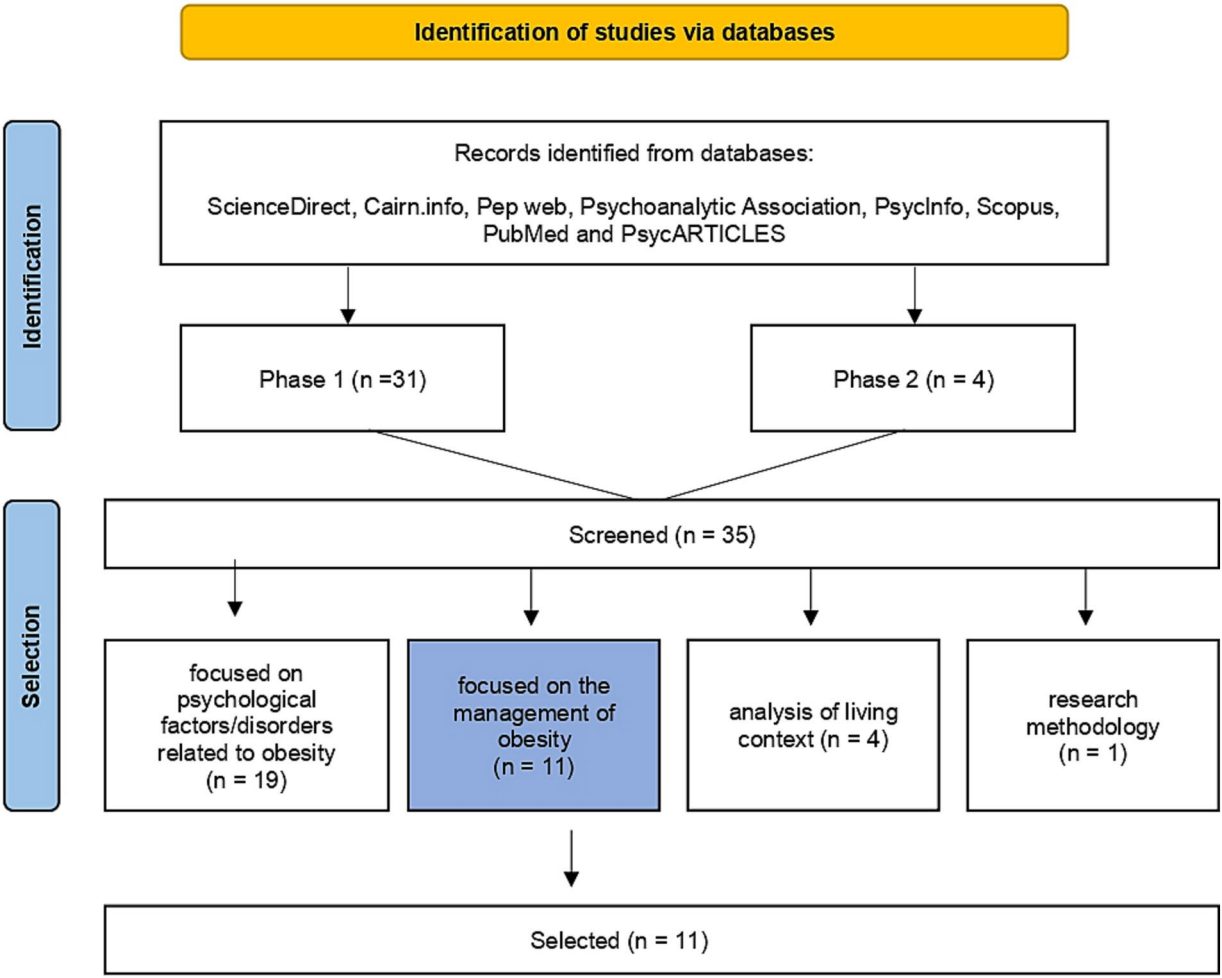
Flowchart for identification and selection of studies.

In summary, at the end of the first phase of research, 31 articles published in the last 10 years were identified, and an additional 4 articles were identified during the second phase. Of those, 11 articles focused on the management of obesity, 19 articles focused on psychological factors/disorders related to obesity, 4 articles focused on analysis of living and context and 1 article focused on research methodology (Figure 1).

There was an average production of about one article per year on the psychological treatment of young adults suffering from obesity, with a publication gap in this topic from 2019 to 2023, with no available findings within this period. This observation highlights the potential for further in-depth research in this field. In light of this observation, the present research was oriented toward the collection and analysis of data gathered from the 11 articles that focused on the treatment of young adults with obesity. This underscores the urgent need for scientific communities, healthcare professionals, and policymakers to recognize the benefits of interventions targeted at this age group in addressing both the individual and collective effects of obesity. Table 1 presents the main characteristics of the 11 studies that have focused on the management of obesity.

**Table 1:** Main characteristics of the 11 studies that have focused on the management of obesity.

Author	Year	*N*	Age range in years	Psychological approaches and techniques	Objectives
Bailey et al. (34)	2017	6	18–25	Semi-structured focus group	Explore preferences regarding the structure and delivery of a weight loss intervention
Batch et al. (28)	2014	365	18–35	Focus group, Cell phone and Personal Coaching interventions; techniques of behavioral self-management	Assess and contrast the effects of a personal coaching intervention and a mobile phone-delivered behavioral intervention on weight loss in young adults
Corsino et al. (25)	2013	33	18–35	Group discussion, nominal technique	Identify factors that may facilitate recruitment of young adults into a weight loss intervention trial
Lanoye et al. (12)	2018	n/a	n/a	Literature review	Provide a foundation for next steps with respect to developing weight management interventions for emerging adults
Lanoye et al. (29)	2016	n/a	n/a	Literature review	Explore young adults, attitudes toward obesity and weight management
LaRose et al. (24)	2019	52	18–25	Behavioral weight loss, in-person, web or hybrid coaching sessions	Determine the feasibility of engaging and retaining young adults in loss weight programs
Lous and Freund (32)	2016	495	20–45	Consultation with general practitioners, interventional group	General practice trial setting to identify predictive factors for weight loss after 1 year among young adults
Juchacz et al. (12)	2021	60	20–30	Psychotherapy group, psychodynamic psychotherapy	Assess the impact of supportive psychotherapy on reducing body weight in young women with overweight and obesity
Robertson et al. (26)	2014	17	18–26	Theory of planned behavior, Semi-structured interviews, Focus group	Explore the specific factors that are relevant to weight control in overweight and obese young adults compared to older adults
Stephens et al. (27)	2015	17	18–25	Focus group	Explore through the opinions of young adults on the use of technology for weight loss
Warschburger and Zitzmann (30)	2019	266	16–21	Cognitive-behavioral group treatment	Determine whether age-specific interventions enhance the efficacy of standard cognitive-behavioral treatment (CBT) provided across age groups in comprehensive inpatient settings

In the subsequent section, we will highlight the core findings from the selected authors in this literature review. We primarily focus on the presented psychological approaches and techniques Additionally, we examine the representation of young adults in the research samples, the objectives behind the interventions, and the strategies by which studies navigated the specific challenges and considerations of this age demographic, especially concerning their inclusion in weight-reduction programs and obesity-related research (Table 2).

**Table 2:** The average number of articles published in the last 10 years on the psychological treatment of young adults suffering from obesity stratified by different approaches.

Approach	*N*
Number of articles that proposed group interventions	9
Number of articles that used a behavior modeling concept	6
Number of articles that proposed a Psychodynamic approach	1
Number of articles that used weight loss during and after the intervention as their primary criterion for evaluation	10
Number of articles that explored young adults’ perspectives on effective weight loss methods	7
Number of articles that examined the benefits of integrating digital tools in weight loss approaches	5

Furthermore, to facilitate a more nuanced understanding of the methodologies employed within these studies, Table 3^3^ has been included, which delineates the strengths and weaknesses of the approaches described, offering an analytical perspective on the robustness and limitations of the research conducted in this domain.

**Table 3:** Strengths and weaknesses of methodological approaches in articles on the psychological treatment of young adults with obesity published in the last 10 years.

Author	Criteria	Description	Strengths	Weaknesses
Bailey et al. (34)	Sample selection	Rural overweight and obese young adult males (YAMs) aged 18–24.	Targeted focus on a specific, underserved demographic.	Limited generalizability to other demographics.	Data collection methods	Participatory design process using semi-structured focus groups.	In-depth engagement with target group for tailored intervention design.	Qualitative approach limits broader statistical application.
Data analysis techniques	Thematic analysis of focus group discussions.	Comprehensive and detailed qualitative insights.	Lacks quantitative data for broader applicability.
Theoretical framework	Focus on personalization and engagement in lifestyle interventions.	Innovative, participant-centered approach.	May lack a broader theoretical underpinning.
Contribution to knowledge	Insights into preferences and needs of a specific group for lifestyle interventions.	Addresses a gap in research for this demographic.	Specific findings may not be directly applicable to other groups.
Batch et al. (21)	Sample selection	Overweight/obese adults aged 18–35, using mobile phones.	Targets a technology-savvy, at-risk demographic.	Limited to specific age group and mobile phone users.	Data collection methods	Randomized controlled trial with interventions delivered via mobile technology.	Innovative use of technology for intervention delivery.	Potential technology-related barriers for participants.
Data analysis techniques	Statistical methods focusing on weight change and engagement with the technology.	Rigorous quantitative approach.	Reliance on self-reported data may affect accuracy.
Theoretical framework	Behavioral interventions delivered through mobile technology.	Integrates modern technology with behavioral health strategies.	May not fully explore the depth of behavioral theories.
Contribution to knowledge	Examines the efficacy of mobile technology in weight loss interventions.	Addresses a gap in the use of technology in health interventions.	Findings may not be generalizable outside the context of technology use.
Corsino et al. (17)	Sample selection	Young adults aged 18–35, overweight/obese (BMI ≥ 25 kg/m^2)	Focused on a specific, underrepresented group in weight loss trials	Limited diversity outside the specified age and BMI range	Data collection	Group discussions using the nominal group technique	Direct feedback from target demographic, structured discussion	Potential for groupthink or dominance by more vocal participants
Data analysis	Thematic analysis of group discussion data	Identification of relevant themes for young adults’ motivations and barriers to trial participation	Qualitative data, subjective interpretation
Theoretical framework	Based on formative research for recruitment strategies	Informed by previous research and tailored to target demographic	Limited exploration of theories beyond the scope of recruitment
Contribution to knowledge	Insights into recruiting young adults for weight loss trials	Valuable information on effective recruitment strategies for a specific demographic	Specific focus on recruitment may limit broader applicability
LaRose et al. (24)	Sample selection	Recruited emerging adults aged 18–25 years, BMI 25–45 kg/m^2^.	Focus on a specific, high-risk demographic for obesity.	Limited generalizability due to narrow age and BMI range.	Data collection methods	Randomized controlled trial using digital and print recruitment methods.	Rigorous experimental design.	Recruitment methods may limit sample diversity.
Data analysis techniques	Analysis using IBM SPSS, focusing on engagement, retention, and weight loss.	Comprehensive statistical approach.	Small sample size may limit statistical analysis power.
Theoretical framework	Behavioral weight loss approach, adapted for emerging adults.	Evidence-based approach tailored to specific demographic.	Specificity may limit broader applicability.
Contribution to knowledge	Insights into behavioral weight loss strategies for emerging adults.	Addresses research gap for this age group.	Pilot nature limits generalizability of findings.
Lous and Freund (32)	Sample selection	Participants aged 18–35 with BMI ≥ 25 kg/m^2^ were recruited from the community and universities.	Inclusive age range and BMI criteria aimed at a diverse young adult population, suitable for the intervention’s target demographic.	Potential bias toward individuals available and interested in weight loss studies, possibly not representative of the wider young adult population with obesity.	Data collection methods	The study used digital platforms for data collection, including online surveys and digital food diaries.	Leveraged modern technology for efficient data collection and participant engagement.	Risk of self-report bias and data inaccuracy in digital entries.
Data analysis techniques	Data were analyzed using logistic regression to understand predictors of recruitment success.	Statistical analysis appropriate for understanding complex relationships between variables.	May not capture the full scope of qualitative data on participant experiences.
Theoretical framework	The study followed a behavioral change theory framework, focusing on lifestyle interventions.	Framework aligns well with the practical aspects of weight management interventions.	May not address underlying psychological issues contributing to obesity.
Contribution to knowledge	The study provides insights into effective recruitment strategies for weight loss trials.	Contributes valuable knowledge on engaging young adults in clinical research.	Focused more on recruitment strategies than the efficacy of the weight loss program itself.
Juchacz et al. (12)	Sample selection	The study selected young women aged 23–25, overweight or obese, ensuring a specific and targeted demographic.	Targeted focus on a specific demographic (young overweight and obese women)	Limited in generalizability due to narrow demographic focus	Data collection methods	Nutrition workshops, psychotherapy sessions, and anthropometric measurements at different stages.	Combination of qualitative and quantitative methods	May lack depth in individual psychological assessment
Data analysis techniques	The study employed descriptive statistics, Student’s t-test, and chi-square test, aiming for robust analysis but could be limited in exploring complex relationships.	Use of established statistical tests for robust analysis	Limited in exploring more complex psychological factors
Theoretical framework	The study integrates psychological and nutritional perspectives, offering a comprehensive approach to weight loss.	Integrative approach combining psychological and nutritional aspects	Potential lack of depth in exploring each aspect thoroughly
Contribution to knowledge	Explores the role of psychotherapy in weight loss, providing insights for both research and clinical practice.	Fills a niche in understanding psychotherapy’s role in weight loss among a specific group	Limited implications for broader psychological research and practice due to the study’s specific focus
Robertson et al. (18)	Sample selection	Included 23 overweight and obese adults, both young and older.	Diverse age range providing insights across different life stages.	Limited generalizability due to small sample size and specific age groups.	Data collection methods	Qualitative approach using semi-structured interviews and focus groups.	In-depth, qualitative insights into individual experiences.	Qualitative data may lack statistical representation.
Data analysis techniques	Thematic analysis with both inductive and deductive approaches.	Comprehensive analysis capturing a wide range of themes.	Subjective interpretation of qualitative data.
Theoretical framework	Application of the Theory of Planned Behavior (TPB).	Theoretical grounding provides a structured approach to behavior.	TPB may not account for all factors influencing weight management.
Contribution to knowledge	Insights into weight management across different age groups.	Enhances understanding of motivational and behavioral aspects.	Focus might not be generalizable to all weight categories.
Stephens et al. (20)	Sample selection	17 young adults aged 18–25. 12 wanted to lose weight, 5 maintain weight.	Appropriate sample size for focus groups. Clear inclusion criteria (age and interest in weight loss).	Convenience sample, not representative. Lacks diversity (mostly women). Selection bias possible.
	Data collection methods	3 focus groups led by single moderator following a discussion guide, with detailed note taking. Audio recordings.	Qualitative method appropriate for exploring perspectives/opinions. Structured discussion guide. Multiple coders (moderator + note taker).	Possibility of moderator bias. No triangulation (observations, etc.).
	Data analysis techniques	In-depth thematic analysis with repeated reading of transcripts, inductive coding, and identification of emerging themes.	Rigorous method allowing important themes to emerge from participants’ perspective. Open coding without *a priori* assumptions.	Analysis done by a single researcher. Risk of confirmation bias.
	Theoretical framework	No explicit theoretical framework. Inductive, exploratory approach.	Fresh perspective without theoretical preconceptions. Discovery of new concepts and ideas.	Lacks grounding in existing literature. Hard to generalize.
	Contribution to knowledge	Original findings on young adults’ perspectives regarding use of technology for weight loss.	Addresses gap in literature. Direct input from target population. Practical implications for intervention design.	Modest contribution given small sample size. Finds results similar to other qualitative studies.
Warschburger and Zitzmann (23)	Sample selection	Adolescents and young adults aged 16–21, with obesity	Targeted a specific age group with a critical health issue	Limited age range, lack of diversity in terms of demographics	Data collection	Cluster-randomized controlled study, BMI and QoL measurements	Structured, objective measurement methods	Potential lack of depth in qualitative data
Data analysis	Statistical analyses using SPSS, ANCOVAs, repeated measures ANOVAs	Rigorous statistical methods, comprehensive data analysis	High reliance on statistical methods might overlook nuances
Theoretical framework	Cognitive-behavioral approach, focus on age-specific interventions	Well-established theoretical grounding, focus on a specific age group	May not account for individual differences beyond age
Contribution to knowledge	Investigates age-specificity in weight loss programs	Addresses a gap in understanding age-specific intervention effectiveness	Findings may not be generalizable beyond the age group studied

### 3.1 Early interventions to accompanying obese young adults

In this section, we will present the epistemologies, techniques, and devices from the field of psychology that have underpinned the discussions in the selected articles regarding treatments offered to young adults with obesity. The collected data indicate which psychological approaches and modes of intervention have predominated in the scientific productions within this field of knowledge. These relevant elements need to be considered to identify the practical and theoretical contributions that Psychology has been generating in relation to the topic of young adults with obesity.

#### 3.1.1 Behavioral modeling as predominant concept

The therapeutic intervention proposed by the majority of the articles was based on behavior modeling concept aiming to increase self-regulation, develop tools for managing stress and motivating weight loss, all in a ‘coaching’ format (24, 26, 28–30, 32), such as: “Important elements of motivational interviewing were used to focus on supporting specific self-efficacy, which means the confidence in own ability to reach a specific goal” (32), and “therefore, young adults may benefit from interventions that focus on self-control and self-regulation, which have been found to be important for many health behaviors” (26).

Cognitive-behavioral principles, including goal setting, self-monitoring, cue-control, and reinforcement strategies, were systematically applied across various stages of the intervention program (30). These principles were employed in psychoeducation sessions, group discussions, video materials, role-playing exercises, individualized worksheets, and “homework” assignments throughout the program, such as: “With a particular emphasis on the enhancement of self-esteem and self-management skills as important individual resources, we aimed to promote long-term treatment success (i.e., stabilization of the attained weight loss during rehabilitation)” (30).

An example of how this approach can be utilized is when, at the end of the health consultation, patients’ resources, and obstacles toward achieving their objectives were addressed and outlined, with their time schedules recorded: “This style of intervention is now called life coaching” (32). The goal of the interventions was to emphasize the bolstering of self-efficacy, signifying the belief in one’s capacity to achieve a particular goal. Therefore, while no specific recommendations were provided regarding food and exercise, general advice focused on the potential benefits of weight loss.

The problem of stigmatization experienced by young adults with obesity, which creates a cycle of stress and weight gain, has been highlighted (29). To address this issue, they propose teaching social skills to manage unsupportive interpersonal situations and fostering the development of autonomous self-regulation for weight loss. These interventions aim to reduce the impact of social control and judgment on individuals. It has been inferred that managing unconscious identifications and references built over the years could be addressed through taught skills (29). In other words, the process of developing identities and self-perceptions, which are shaped unconsciously over time, could be influenced, and transformed by teaching specific skills.

Robertson et al. (26) showed, when implementing the Integrated Behavior Model, that despite several participants expressing a strong sense of control and confidence in their ability to adopt weight-loss behaviors, this belief contradicted their real-life experiences, as they encountered significant challenges in initiating and sustaining these behaviors (26). This observation is evident from the fact that most participants disclosed their prior attempts at weight loss; nonetheless, they also acknowledged regaining the lost weight. According to these findings, the discrepancy between knowledge and practical skills may play a role in the participants’ history of unsuccessful weight-loss attempts, aligning with existing literature indicating that although knowledge is necessary for behavioral change, it is not the sole determinant of successful outcomes.

This sheds light on the underlying complexity of the human relationship with food. Eating is not merely a biological act to satisfy a physiological need; in other words, ingesting food is not solely a matter of survival. It also involves the role that food represents in a person’s psychological involvements. In essence, behind a meal lies the significance of that moment in the individual’s history, along with the impulses or compulsions that eating may or may not trigger. Consequently, unconscious pathways can be activated, even if the person imposes measures of control or rational commands.

These attempts are likely to fail if there is no concurrent work with the unconscious representations surrounding the act of eating, which are unique and specific to each individual (26).

#### 3.1.2 Psychodynamic approach as an epistemological contribution

Among 11 studies analyzed, it was further noted that only one article refers to psychodynamics as a relevant epistemological contribution to the management of obesity in young adults. It is important to clarify Psychodynamic psychotherapy is a therapeutic approach rooted in psychoanalytic theories, which were originally developed by Freud (33). This form of therapy focuses on the psychological forces that underlie human behavior, feelings, and emotions, and how they might relate to early experience. It emphasizes the exploration of the unconscious mind and seeks to understand how these unconscious processes influence current behavior and relationships. The approach involves examining unresolved conflicts and symptoms that stem from past dysfunctional relationships and manifest themselves in the present. Psychodynamic therapy aims to uncover these past dynamics, understand them, and integrate this understanding with current behavior.

The authors start from the initial point that in addition to excessive food consumption, a psychological component may contribute to the development of obesity (19). In today’s environment, which encourages obesity, food transcends its role as a mere means to satiate hunger. It becomes an essential surrogate element in mitigating deficiencies not linked to nutrition, such as the need for emotional connection, for instance.

Data regarding the effectiveness of cognitive-behavioral therapy (CBT) and behavioral therapy (BT) for obesity treatment are generally inconclusive due to the heterogeneity of the conducted studies (19). A permanent transformation in dietary habits and lifestyle is necessary to achieve satisfactory long-term weight loss. However, this may prove inadequate in preventing weight regain if underlying eating disorders remain unaddressed. Therefore, it has been suggested that psychodynamic psychotherapy could be a beneficial supplement to traditional obesity treatment approaches, since it aims to assist patients in dealing with profound psychological issues, such as “the personal development of the participants, the acceptance of their own bodies, strengthening their egos, and the self-experience of their emotions” (19).

It is recognized that studies on the use of psychodynamic psychotherapy in obesity treatment are limited, but they have demonstrated high effectiveness when combined with a healthy diet, physical activity, and dietary counseling. Some studies on the use of CBT and BT as supports for obesity treatment have shown disappointing results in terms of long-term weight loss (19).

#### 3.1.3 Group interventions in obesity management

Seven studies proposed group interventions; however, there was no discussion regarding the effectiveness of group interventions in managing young adults (12, 24–28, 30, 32, 34).

Research conducted by psychodynamic psychotherapists approached this issue: “The study shows that the strategy integrating a diet with a dietary consultation and regular psychotherapeutic group sessions proved to be the most effective in reducing body weight and body fat (…) when compared to the other strategies tested (diet + exercises, diet + exercises + dietetic consultations, and diet + exercises + psychotherapy)” (19). However, although the benefits of group psychotherapeutic processes are acknowledged, the study does not justify or explicitly differentiate the preference for group intervention over an individual approach.

With the implementation of a weight loss project in a specific clinic for young adults, it was highlighted that due to their shared environment, participants had abundant opportunities to partake in mutual learning and experiential exchange with peers of the same age (30). This emphasizes the understanding that group work would foster experiential exchanges as “(…) social exchange was likely to occur even beyond the confines of the therapy sessions” (30).

Focus groups were also used, which covered topics such as overweight and obesity in young adults, diet and exercise habits, counseling strategies for weight loss or weight maintenance, and the use of Smartphone technology for weight loss (25). However, the choice of the group approach was not elaborated upon and justified, acknowledging it as a treatment device for psychological elements. In another study, a special approach was implemented, combining in-depth semi-structured focus groups and individual interviews, without the authors developing its specificities (26).

In the following section, we will explore the authors’ perspectives on the representation of young adults in research, emphasizing the imperative for both programs and researchers to tailor their approaches to the distinct distinctions of this demographic.

### 3.2 Representativeness of young adults with obesity in psychology research

From the reading and analysis of the 11 articles selected and included in the “psychological treatment” category, it can be observed that there is a consensus (19, 24, 25, 28–30) on the fact that research and specific interventions for the young adult age group are underrepresented. As some authors point out, *“*Great advances have been made in behavioral weight loss interventions for children, adolescents, women, and racial/ethnic minorities; however, one high-risk group that has been largely overlooked is young adults” (29) and “However, young adults ≤35 years of age are underrepresented in behavioral weight loss trials, limiting generalizability of the findings to this age group” (25).

The fact that they are underrepresented in research reflects directly in a lack of interventions specific to this subgroup, receiving treatments identical to those offered to adults. Standard intervention approaches for adults often fail to address weight-promoting developmental concerns and risk factors that are specific to this age group. This may contribute to their inadequacy to effectively address the needs of this population (19). Gokee-LaRose et al. (24) adapted evidence-based behavioral weight loss programs for emerging adults, incorporating psychological needs outlined by the Self-Determination Theory. The study observed favorable outcomes related to weight loss. Participants in the web-based 3-month lifestyle intervention, which included optional community sessions, exhibited greater weight loss compared to those engaged in either web-based or face-to-face interventions similar to adult weight loss programs.

In addition to the essential distinction between interventions for young adults and adults, the significance of equally addressing the unique characteristics of the subgroups emerging adults and young adults was emphasized (19). By encouraging emerging adults to identify priorities and assume individual responsibility within weight control programs, we would be effectively guiding them to navigate the transitional gap between these two developmental stages. To exemplify the relevance of a specific approach to this age group, the authors observe that the emerging adults may opt for alternative approaches to address weight management, such as seeking guidance from their primary care physician, enrolling in commercial weight loss programs, or participating in behavioral weight loss trials (19). However, these options appear to be unattractive, inaccessible, or unfamiliar to many individuals in this age group, as evidenced by their limited presence and participation in such settings.

Recognizing the imperative of aligning research methodologies to the particularities of this target population, we will subsequently address the core objectives that underlie these studies.

### 3.3 Weight loss as a unique aim

To quantify the outcomes of an intervention targeted toward young adults with obesity, researchers have elected weight loss during and after the intervention as their primary criterion for evaluation (12, 24–30, 32, 34). This choice reflects an emphasis on tangible, physical metrics, potentially underestimating the multifaceted nature of obesity and its psychological implications. It may be inadvertently downplaying the importance of acknowledging obesity not merely as a standalone physiological condition, but as a symptom that plays a structuring role in the individual’s life. In other words, despite causing distress, obesity often serves as a stabilizing function, acting as a containment for emotions, traumas, and sufferings that would otherwise overflow and potentially lead to more intense subjective disorganization.

Researchers who proposed the psychodynamic group approach in the follow-up of young adults also measured the effectiveness of this intervention by considering weight loss as one of the evaluation metrics (12). The same can be observed in the research employing cognitive-behavioral treatment intervention as their approach: “Therefore, targeted weight loss interventions and hence a development-sensitive approach seem crucial” (30) and “There is a need to understand the types of interventions that would target college students who are interested in losing weight” (27).

However, contrary to the findings of these studies that emphasize weight loss, other studies have observed that young adults seek programs with broader goals (19). They expressed a preference for an approach that emphasizes the overall benefits of lifestyle changes, such as increased energy, improved fitness, and enhanced health, rather than solely focusing on “weight loss.” In other words, besides the potential drawbacks associated with weight-centered interventions, which will be further explored, these approaches may not fully align with the preferences and expectations of the targeted young adult population.

### 3.4 Specific points to consider in the development of a management program adapted to young adults

Some of the included articles focused on exploring the treatment perspective for young adults with obesity by considering the specificities of this population in their participation in weight loss programs. The following points were developed: considerations for efficient recruitment based on the characteristics of this population, suitable methods/techniques for weight loss in this age group, and the integration of technology in interventions with young adults.

#### 3.4.1 Evaluating the efficacy of intervention and recruitment models

One study aimed to identify which model of intervention and recruitment would be most effective for this age group (either through physical, virtual, or hybrid meetings) (19). The effectiveness was measured by weight loss during and after the study. As for recruitment, the first question was to determine which elements would tailor to the interests of this target audience to maximize their engagement in studies and weight loss programs. In fact, strategies such as social media promotion, timing adjustments, offering health and weight loss benefits, providing dietary and exercise information, and offering incentives (monetary rewards, gym access) are considered. Themes emphasizing benefits and convenience aimed to dispel misconceptions about research among the public and flexibility with scheduling and timing were proposed (24, 25).

According to Lanoye et al. (19) and LaRose et al. (24), combining technology with in-person activities shows promise in young adult populations. A pilot trial comparing technology-only, in-person-only, and hybrid delivery of behavioral weight loss interventions found that the hybrid approach was the most effective in promoting engagement and achieving clinically significant weight loss outcomes among 18-25-year-olds.

It is essential to emphasize the apparent dichotomy between the need for autonomy and accountability in interventions offered to young adults. It was observed that an approach that supports autonomy improved engagement and retention in a weight loss trial for emerging adults (19, 24).

The specificity of young adult men has also been highlighted (29). To potentially engage more young male participants, intervention programs could adopt targeted advertisements emphasizing “fitness” rather than “weight loss.” This is because young men who express dissatisfaction with their weight are more inclined to focus on gaining weight or achieving a more muscular physique, rather than aiming to lose weight.

To adapt the intervention method to the target audience, it was also mentioned the importance of addressing time management and stress management to enhance engagement of young adults in a weight loss program (29). This entails considering flexible and convenient scheduling, and potentially incorporating the use of mobile and web platforms to meet their needs. Moreover, it should be noted that interventions should also address interpersonal factors, such as affiliation, competition, and social recognition, as they play a significant role in guiding young adults’ behaviors and choices. Lastly, it should be noted that this age group is more susceptible to social influences, with motivations primarily guided by peer recognition rather than health concerns (24, 26, 29).

In contrast to these findings, in a study with focus groups conducted with 33 young adults, health was prioritized over appearance when it came to weight concerns (25), despite a recent report suggesting otherwise. The study participants ranked health as the most significant factor related to weight. The researchers hypothesized that this awareness may be a result of the growing public understanding of the association between unhealthy weight and the development of chronic conditions such as hypertension and diabetes.

Finally, regarding the mode of participant follow-up (in-person, virtual, or hybrid), a hybrid approach, combining both in-person and virtual elements, showed promising results (24). The hybrid approach, delivered primarily through the web, achieved comparable engagement and better weight loss outcomes than the face-to-face program, even with adapted content and individualized check-ins. The hybrid condition outperformed the web-only arm in terms of engagement, retention, and weight loss, indicating a specific factor in the hybrid approach that promoted engagement beyond technology-based delivery. Therefore, the increased emphasis on promoting autonomy and providing choices likely led to improved engagement in the hybrid arm.

A study conducted with rural overweight and young adult males with obesity observed, however, that for this specific audience, there is no difference between face-to-face and virtual meetings, implying that both formats are equally effective: “Virtual versus physical face- to-face conditions are not necessarily considered different by young adults” (34). Thus, it would be crucial to ensure that the messages sent by the coaches are personalized.

#### 3.4.2 Perspectives on weight loss methods among young adults

Several studies have explored young adults’ perspectives on effective weight loss methods (19, 25, 27–29). The main elements identified were physical activity, dietary changes, social support, medical interventions, and self-control. As it was found in one of the studies: “Participants highlighted physical activity, dietary intake, social support, medical intervention, and taking control (e.g., being motivated) as the best weight loss strategies” (28).

As an illustrative point, young adults demonstrate a admirable openness to modifying their routines, with a specific focus on physical activity and nutrition. It’s noteworthy that motivations for engaging in physical activities among this group are predominantly rooted in interpersonal and societal considerations, such as the communal aspect of sports. The inclination toward embracing the teachings of mindfulness skills holds promise in sharpening their acuity regarding dietary habits. Concurrently, a pronounced propensity has been observed among these young adults to collaborate with nutritionists, ensuring their dietary patterns align more closely with healthier nutritional standards (29).

Thus, psychological support is not considered as a potential approach to assist young adults in managing the distress associated with obesity and this stage of their lives. Notably absent from these identified strategies, however, is the domain of psychological support. This absence is somewhat bewildering, considering the intertwined nature of psychological well-being and holistic health. While physical interventions are undoubtedly paramount in addressing obesity, the emotional and psychological undercurrents accompanying this condition, especially during the transformative stage of young adulthood, cannot be understated.

#### 3.4.3 Digital tools integration in weight loss accompanying among young adults

Some studies have examined the benefits of integrating digital tools in weight loss approaches among the young adult population (24, 27–29, 34). As reported, “The demand for an individualized program was high. Young adults wanted an application that is specific to their height, weight, gender, age, and weight loss goals” (27) and “Findings suggest that to appeal to this population, programs should be brief and delivered using a hybrid format with some in-person contact augmented with a technology platform” (24).

Regarding the use of technologies in weight loss programs, messages should include individual feedback, and that motivational and positive feedback messages are preferred (27). Additionally, participants seem to desire an all-inclusive application for their weight loss journey.

On the other hand, a study aimed at scrutinizing the specificities of young adults, indicated that, although the use of technology was appealing, exclusively relying on technology-based modalities for intervention delivery had the potential to diminish perceived accountability and to expedite disengagement over time among young adults (19). The authors of another study also acknowledged that “It is worthwhile to consider new approaches to weight management in young adults that are designed to fit their lifestyles and circumstances. One such approach is use of mobile phone technology for delivery of a behavioral intervention” (28).

## 4 Discussion

In this following section, we will undertake a comprehensive examination of the results elucidated in the prior segment. The structure of our discourse will unfold as follows: Initially, we shall explore the importance given to psychological support and the subjective intricacies compared to the predominant aim of weight reduction. This aim will then be critically assessed considering the identified constraints within our surveyed literature, emphasizing the invaluable insights that both group-centric methodologies and psychodynamic orientations can provide, emphasizing a perspective that transcends mere weight-focused objectives. In our concluding remarks, we will pinpoint prospective trajectories for emergent research efforts to further delineate the young adult demographic, considering that there is still no established consensus among researchers regarding its definitive categorization.

### 4.1 The role of psychological factors in obesity treatment for young adults

The points raised so far suggest that the interventions proposed by the current research primarily focus on weight loss, without adequately addressing the psychological and emotional factors that may influence the development of obesity. Therefore, there seems to be a lack of approaches aimed at exploring in-depth the foundations of the relationship between young adults suffering from obesity and their links to food, to self-esteem, and to family history. In other words, although the harmful effects of a culture that judges and stigmatizes a segment of society for not conforming to the demanded image standard are recognized, the focus of the majority part of the research remains on weight loss as the primary aim.

It is essential to recognize that the obese body can serve as a defense mechanism or coping mechanism for emotional challenges (35–39). Thus, solely concentrating on weight loss without considering this symbolic dimension may leave unresolved emotions without proper channels, leading to additional psychological difficulties (3, 5, 10, 29, 38, 39).

There is no denying that reducing body fat can lead to a decrease in obesity-related secondary diseases. However, it is crucial to challenge the subjective effects, both on the participants and within the cultural discourse, of interventions solely aimed at weight loss. It is pertinent to consider that such interventions may inadvertently reinforce prejudices and stigmas, which are already sources of stress, anxiety, and, consequently, weight gain. Therefore, it becomes essential to explore alternative approaches that address the psychological and emotional well-being of young adults with obesity, rather than solely focusing on weight reduction.

Rather than exclusively focusing on symptom remission through weight loss, a more foundation-oriented approach may offer prospects for long-term transformation. For example, by examining relationships with the environment, self-identification, social and family influences, it is possible to become aware of certain unconscious factors that influence the relationship with food, which are often unknown to the individual themselves. Elaborating on one’s own history, relationships, and how we integrate social demands, including expectations surrounding an image shaped by slim and toned bodies, is a pathway to both questioning rigid and harmful responses and building new perspectives and ways of positioning oneself in the world.

Perspectives rooted in psychodynamic theory interpret weight gain and the development of an obese body as an unconscious response of the individual to some invasive trait in their family dynamics and/or a traumatic event (40–42). In other words, it is through this body shaped over the years that they position themselves in their relationships and construct their self-image. Eating, even compulsively, might be the pathway found by the individual to give meaning to some unelaborated issue that persists and demands to be mentally “digested” (40, 41, 43). This act, therefore, is an attempt to restore balance in the body–mind system.

In this sense, we may assume that approaches that do not take these factors into account tend to limit their effort to educational and adaptive interventions, which may lead to situations where the desire to control eating does not prevent further loss of control and overeating.

Based on the results of our review, it can also be inferred that psychological aspects associated with obesity are still culturally neglected (19, 24, 27, 29) or even denied. Despite obesity being recognized by the medical field as a complex and multifactorial disease, where emotional and psychological factors are acknowledged, the participants showed disregard for a psychotherapeutic approach in weight loss. This finding may indicate the lingering prejudices surrounding the lack of psychological support. In other words, interventions involving diet, physical activity, medications, and surgery are more socially accepted and less stigmatized than acknowledging the influence of psychological elements, which we are driven by and do not control.

### 4.2 Gaps and limits in the current literature

#### 4.2.1 Underrepresentation of group approaches in obesity interventions

In this literature review, one of the primary objectives was to explore psychological treatments, as well as the treatment approaches and perspectives targeted at young adults. A notable limitation within the surveyed literature was the lack of emphasis on group interventions as a facilitative measure in treating the psychological distress linked to obesity.

Studies utilizing group methodologies have not addressed these groups as a facilitating device for specific work with young adults. Consequently, this approach remains underexplored and under-analyzed compared to existing literature on groups with adolescents.

Therefore, it appears that group-based care, aimed at providing psychological support to young adults, has not received adequate attention in publications over the past decade. The group, as a mediating device, has the potential to strengthen verbal expression, projection effects, and identification effects, which are beneficial for the emergence of unconscious aspects.

However, as observed in our literature overview, the studies that utilized groups did not discuss the peculiarities of groups as a special feature, suggesting that the group would only act as a means of exchanging experiences among participants and/or support for implementing group dynamics. This perspective overlooks the work of theorists, like Rene Kaës, who have developed the concept of the group as a device capable of expressing a group unconscious, rather than solely an individual one (44). This group’s unconscious is shaped by the interactions, fantasies, and conflicts within the group, creating a unique and powerful force that influences the thoughts, feelings, and behaviors of its members.

By recognizing the group as a dynamic entity with its own unconscious, we can gain a deeper understanding of how group members influence each other and how the group, as a whole, impacts individual experiences and behaviors. In the context of obesity treatment, this perspective opens new possibilities for addressing the psychological and emotional aspects of young adults with obesity within a group setting.

As an example, some studies have used group therapy as a therapeutic device in their processes. One study explored the interactions between the phenomenon of immersion and the perceptual-hallucinatory activity from a psychodynamic perspective, conducting a therapeutic group context using virtual and digital mediation (45). Another research aimed to compare the effectiveness of group therapy and individual therapy in promoting weight loss in obese children and adolescents (46). Fifty patients were involved in this study, with individual therapy provided in the first year and group therapy in the second year. The results showed that group therapy was more efficient, as only 10% of patients experienced significant weight gain. Therefore, group therapy was found to be more effective in inducing weight loss in this age group.

A therapeutic group intervention for adolescents was administered to a cohort of approximately 30 young girls, aged 13 to 17 years with overweight (47). The therapeutic approach created a contained setting, enabling them to interact with a group mirror where individual differences were lessened. Within this group context, the participants were empowered to perceive themselves as beautiful. This recognition from their peers stimulated their desire for self-empowerment and self-nurturing. These are just a few studies mentioned to illustrate the diversity of research in which the group therapeutic approach is used as a facilitating and enhancing mean for therapeutic effects.

#### 4.2.2 Scarce representation of psychodynamic approaches

The literature review also shows that the majority of articles were based on behaviorism, suggesting that the psychodynamic approach is underrepresented. This does not necessarily mean that the psychodynamic approach is not being used directly with patients in clinics and hospitals, but rather that scientific publications from this field of knowledge may be less frequent.

The specificity of this developmental period and its potential for conducive changes can be explored within the domain of psychodynamics. Psychological treatment of obesity is not necessarily culturally perceived as an integral part of treatment, possibly due to the predominant association of this condition with the realm of biology and physical health. Nevertheless, the psychological perspective, including the psychodynamic approach, illuminates the significance of subjective factors in the genesis of psychosomatic disorders, questioning the inclination to dichotomize the relationship between the body and the mind.

In this developmental phase, it is not so much the biological transformations that prevail, as in puberty, but the subjective effects of these transformations in the face of new contexts, relationships, and social demands (48). The search to find one’s place in society (49), with the expectations and pressures involved in this quest, such as social status, finding fulfilling and suitable work, the desire and obligation to engage in a romantic partnership, the fears and disappointments in love encounters, and the responsibility that comes with the long-awaited autonomy, are factors that occupy a significant space in the subjective lives of young adults, sometimes becoming sources of anxiety and distress. Psychodynamic therapy serves as one of the possible avenues to assist them in navigating this period laden with social demands, by listening to, considering, and working through their unique desires, which may at times be discordant with these demands and thus difficult to embrace.

Therefore, the treatment provided by psychodynamic therapy aims to accompany the process of recognition, choice, and acceptance of a life path. This process is not about age but about the capacity to take a stand independently of preconceived expectations. Family and the social context of friends and colleagues have a powerful influence on the chorus of expectations, making it tough sometimes to maintain one’s own voice in these relationships.

Elaborating on the position we occupy in each of our relationships opens perspectives for learning to how do we deal with feelings, emotions, dreams, and desires. In doing so, we discover new ways to channel frustrations and anxieties that previously found painful exits, such as compulsive eating (46). Psychodynamic therapy thus presents itself as a potent pathway for listening to this subjective life stage, particularly as a period of decision-making, self-discovery, and asserting one’s own voice.

Regarding the limitations of this literature review, it is worth mentioning the choice of English and French as the languages for the selected keywords. While these languages encompass a significant portion of scientific production, they do not cover all languages, such as Portuguese and Spanish, which might have relevant contributions in the field. Additionally, this review focused primarily on research with obesity as the central topic, thereby narrowing the scope to exclude most studies in psychology concerning eating disorders and overweight. Consequently, the relatively low number of articles found over a ten-year period, totaling 11 articles, does not encompass the entire scope of studies on overweight and eating disorders in young adults. Some studies, such as those mentioned for illustrative purposes (49–51), may have been excluded due to these criteria.

### 4.3 Gaps and limits of this study

In recognizing the nuanced nature of psychological dimensions related to obesity, we emphasize the importance of categorizing these aspects based on BMI indices and accounting for gender distribution. These variables are acknowledged as pivotal factors that may exert substantial influence on both psychological and physiological responses to interventions targeting obesity. It is essential to note, however, that our current article selection criteria did not incorporate these variables, rendering our chosen literature unsuitable for an exhaustive examination of such intricacies.

This oversight marks a significant limitation in our study, highlighting the necessity for future research to explore these particular distinctions in depth. As a result, a thorough examination of the interactions among psychological factors, BMI indices, and gender distribution is essential to more clearly understand their combined impact on effectively managing obesity in young adults. Recognizing this gap encourages further investigations, providing critical insights that can refine treatment strategies for obesity and deepen our comprehension of its complex causes.

### 4.4 Strengths and perspectives

Upon conducting this literature integrative review, it became evident that there is a lack of consensus regarding the definition of the age range for young adults, with some authors including emerging adults in the category. When grouping young adults aged 18–25 with those aged 25–35, we fail to acknowledge the specific factors that characterize each stage. Emerging young adults go through a period of greater instability and transformation, experiencing difficulties in establishing responsibilities and autonomy.

As previously discussed, this transformation phase, while presenting challenges, can also be explored for its potential if we acknowledge the benefits of a still-developing ground. Where the structure retains a degree of plasticity, there is also an observable increased adaptability to restructuring. This warrants hope that interventions during this period can be effectively established and have lasting impacts in subsequent years. This, therefore, underscores the importance of directing more research and investment toward meeting the individual and collective needs of this particular group.

Future research possibilities equally indicate the exploration of objectives transcending the conventional focus on weight loss, pivoting instead toward an understanding of the psychological underpinnings of body image, of obesity and the psychosocial determinants influencing eating behaviors. Accordingly, emerging studies are invited to harness the power of group-based methodologies. Yet, this is not a mere call for employing group dynamics in a traditional sense; rather, it advocates for the deployment of such setups as a conduit for eliciting and addressing unconscious elements. In doing so, we posit that these methodologies can serve as catalysts for the deeper psychic processes that warrant scholarly attention.

Further research must investigate these particularities in greater depth, providing a foundation for subsequent studies to build upon and establish a consensus in the scientific field. As a result, new research and public policies can be directed toward addressing the specificities of these life stages, leading to more significant findings and ultimately promoting targeted well-being for this population.

### 4.5 Prospective research pathways in psychological approaches to obesity in young adult

There is an evident gap in longitudinal research regarding the effectiveness of psychodynamic psychotherapy for obesity, specifically in the young adult population. Future studies should aim to evaluate the enduring effects of psychodynamic therapy on weight maintenance and the psychological well-being of young adults, tracing the long-term trajectory post-intervention to ascertain the permanence of therapeutic outcomes.

The role of group interventions tailored for young adults merits further investigation. Research should focus on how these group settings facilitate not only the sharing of experiences but also the revelation and working through of unconscious drivers of obesity. This exploration could unveil pivotal dynamics in the treatment of young adult obesity.

The integration of digital tools in psychological approaches for young adults dealing with obesity is an emerging frontier that warrants rigorous exploration. Studies should assess how digital platforms can be harnessed to augment traditional psychological therapies, enhancing accessibility and continuity of care. This research should also examine the unique challenges and benefits of digital interventions for young adults.

Comparative studies are crucial to discern the relative efficacy of individual versus group psychodynamic therapies in the context of young adult obesity management. These studies should be designed to understand which therapeutic settings are most effective for young adults, taking into account their particular developmental challenges and social contexts.

By proposing these specific research directions, this section underscores the necessity for a concerted effort to develop and refine psychological therapies that resonate with the unique needs of young adults facing obesity. The aim is to foster a more sophisticated and empirically grounded psychological approach that can address the complex interplay of factors contributing to obesity in this age group, ultimately leading to more nuanced and effective treatment paradigms.

## 5 Conclusion

Based on this literature review, we have identified key insights into how psychological studies approach treatments for young adults with obesity. Regarding interventions targeting the unique characteristics of this group, the adoption of hybrid group interventions, which blend virtual and in-person components, serves the purpose of catering to the participants’ specific needs and enhancing their active involvement in the intervention process.

Regarding therapeutic approaches, the psychodynamic approach appears less prevalent in scientific literature than the cognitive-behavioral approach. However, the advancements and benefits of the cognitive-behavioral approach in interventions are widely recognized. It is essential to acknowledge that the psychodynamic approach can also contribute to the social domain by providing a distinct perspective on symptoms, offering an approach less focused on immediate adaptation, and aiming for long-term outcomes. In conclusion, psychodynamic therapy aims to facilitate a process of reconstructing the individual’s relationships and choices, emphasizing a gradual and lasting transformation. By attentively listening to the individual’s desires and unique voice, the therapy delves into the fundamental aspects, fostering enduring changes in the individual’s life.

With respect to the methodologies adopted in the studies, there is a notable lack of depth in exploring the group modality’s capacity to bring forth and articulate unconscious elements. This presents a significant opportunity for further research and advancement in the field.

Finally, it is imperative to recognize the importance of addressing obesity in young adults during this under-construction period, as early interventions can have a lasting impact on their overall health and well-being throughout their adult lives. Continuous work with this population can significantly impact public health by reducing the trajectory of weight gain and obesity at both the individual and medical cost levels. In this way, turning our attention to this life stage will provide individual and collective gains that should be considered in future research.

## Author contributions

RA: Conceptualization, Formal analysis, Investigation, Methodology, Writing – original draft, Writing – review & editing. HP: Formal analysis, Methodology, Writing – review & editing. DD-S: Conceptualization, Formal analysis, Investigation, Methodology, Writing – review & editing.

## Footnotes

^1^It is relevant to highlight that there is no consensus regarding the age range corresponding to the period referred to as young adulthood. Some authors (12, 17, 26–28) classify this life stage between 18 and 35 years, while others (9, 24, 29, 30) propose a subdivision between emerging adults, aged 18–25, and young adults, aged 25–35. For these authors (12, 24, 29, 30), it is important to differentiate emerging adults as they experience distinct transitions and instabilities compared to young adults. We will adopt the age range of 18–35 years as a reference, acknowledging, however, that there are particularities that differentiate the life stages of those who are still in university from those who are already in the workforce.

^2^DINAMO (Towards a digital device for the support of young adults suffering from obesity) developed in partnership between PSInstitut, University of Strasbourg and Strasbourg University Hospital.

^3^In constructing Table 3, our inclusion criteria necessitated the focus on empirical studies that directly engaged with the application of interventions within clinical or experimental settings. Consequently, the contributions by Lanoye et al. (30) and LaRose et al. (25), while invaluable to the literature, were not incorporated into this table due to their methodological framework being that of literature reviews.
